# Association between weight/body mass index and insulin dose requirements in gestational diabetes: a retrospective cohort study

**DOI:** 10.1136/bmjdrc-2026-006285

**Published:** 2026-10-13

**Authors:** Sinziana Turlea, Afnan Randhawa, Lama Almohtasib, Vincent Simpson, Isabelle Kathryn Mayne, Andrew Peter McGovern

**Affiliations:** 1 University of Exeter Medical School, Exeter, UK; 2 North Devon District Hospital, Royal Devon University Healthcare NHS Foundation Trust, Exeter, UK; 3 Royal Devon and Exeter NHS Foundation Trust, Exeter, UK; 4 Royal Devon University Healthcare NHS Foundation Trust, Exeter, UK

**Keywords:** Diabetes, Gestational, Insulin, Pregnancy, Body Mass Index

## Abstract

**Introduction:**

Gestational diabetes mellitus (GDM) is associated with adverse maternal and neonatal outcomes when tight glycemic control is not achieved. Insulin therapy is frequently required to manage fasting hyperglycemia; however, optimal initiation and titration remain challenging within the short treatment window. Weight-based approaches are commonly used to guide insulin prescribing in clinical practice. This study aimed to evaluate whether baseline clinical characteristics, including pre-pregnancy weight and body mass index (BMI), predict insulin requirements in women with GDM.

**Research design and methods:**

We conducted a retrospective cohort study of women with GDM managed using a rapid insulin titration protocol at our center between 2022 and 2023. Baseline demographic and clinical characteristics, including pre-pregnancy body weight, BMI, and oral glucose tolerance test (OGTT) results, were used to predict the initial insulin dose required to achieve glycemic targets and the maximum insulin dose required during pregnancy.

**Results:**

Insulin therapy was initiated in 58 women. Marked interindividual variability was observed in both the insulin dose required to achieve glycemic targets and the maximum insulin dose administered. Regression analyses demonstrated poor predictive performance of pre-pregnancy weight and BMI to reliably predict insulin requirements, as reflected by low R² values: initial insulin dose required to achieve glycemic target using weight (R²=0.61) or BMI (R²=0.58) and maximum insulin dose required using weight (R²=0.30) or BMI (R²=0.36).

**Conclusions:**

Pre-pregnancy weight and baseline BMI have limited utility in predicting insulin requirements in women with GDM. A fixed starting dose with rapid glucose-based titration may represent a more robust way to achieve individualized insulin dose requirements in GDM.

**WHAT IS ALREADY KNOWN ON THIS TOPIC**

- Predicting insulin requirements in gestational diabetes mellitus (GDM) is challenging.
- Measurements such as pre-pregnancy weight and body mass index (BMI) are commonly used to guide insulin dose despite minimal supporting evidence.

**WHAT THIS STUDY ADDS**

- Pre-pregnancy weight, BMI, and oral glucose tolerance test results have poor correlation with insulin requirements in GDM.

**HOW THIS STUDY MIGHT AFFECT RESEARCH, PRACTICE OR POLICY**

- Weight-based or BMI-based dosing should not be relied on in GDM management. Glucose results-driven insulin titration offers a suitable alternative.

## Introduction

 Gestational diabetes mellitus (GDM) is common in pregnancy and associated with maternal and fetal complications. The Hyperglycemia and Pregnancy Outcomes study (HAPO) demonstrated gestational hyperglycemia is associated with pre-eclampsia, premature delivery, babies born large for gestational age, need for interventional delivery, shoulder dystocia, birth injury, neonatal hypoglycemia and hyperbilirubinemia, and need for intensive neonatal care.^1^ In addition to lifestyle advice, insulin is commonly required for management. Given that the treatment window is short, early insulin initiation and titration are essential to quickly optimize glycemic control and reduce the risk for these maternal and fetal complications.

Being able to accurately predict insulin requirements could improve early glycemic control in GDM. Clinical approaches to initial insulin dose selection and insulin titration vary very widely among hospitals,^2^ and across guidelines, with several current protocols recommending using a pre-pregnancy weight-based or body mass index (BMI)-based insulin dose selection. However, few comparative studies have evaluated different approaches to insulin dose selection and titration,^3^ and it remains unclear whether weight or BMI are accurate predictors of insulin requirement in GDM. Therefore, there is a need for an evidence-based approach to insulin dosing in GDM, which is essential to prevent complications and minimize care variation.

We used real-world insulin dose data to develop predictive models of insulin requirements in GDM and explore whether weight, BMI, and other clinical parameters can be used to predict insulin requirements in GDM.

## Method

### Study setting and clinical algorithm

We performed a retrospective observational cohort analysis of women diagnosed with GDM and managed in our specialist joint obstetric and diabetes antenatal service between 2022 and 2023. We used a standardized, patient-led, rapid basal insulin titration protocol for the management of GDM, which we have previously described.^3 4 5^ All women diagnosed with GDM were provided with dietary and lifestyle support at diagnosis and throughout follow-up, with pharmacological therapy initiated in cases of persistent hyperglycemia.

In brief, the insulin titration protocol involves initiating nighttime basal insulin for all women with fasting blood glucose >5.3 mmol/L despite dietary and lifestyle advice, starting at an initial dose of 14 units daily and subsequently titrated daily by patients according to their fasting capillary glucose values. Women are advised to increase their basal dose by 4 units each day following a fasting glucose reading above target ≥5.0 mmol/L. They are advised to reduce their insulin dose if their fasting glucose was <4.0 mmol/L with hypoglycemic symptoms. If fasting glucose targets are achieved, then women are advised to keep their insulin dose the same until it goes out of range again, at which point they can continue dose titration in the same way. This insulin management algorithm works in a manner very similar to a physiological homeostatic system, and its use provides a unique opportunity in GDM to understand the insulin requirements of women throughout pregnancy. Metformin and/or short-acting insulin are used for elevated postprandial glucose in accordance with patient preference (after discussing risks and benefits of both options and the unknown effects associated with transplacental passage of metformin) and the degree of postprandial hyperglycemia. When needed, short-acting insulin is titrated by regular (weekly or twice weekly) reviews by a diabetes dietician, diabetes specialist nurse, or diabetologist alongside the patient-led basal insulin titration.

### Cohort definition and data collection

Women were included in the study if they had a diagnosis of GDM (based on the UK National Institute for Health and Care Excellence diagnosis criteria),^6^ and complete data from an oral glucose tolerance test (OGTT) baseline and 120-minute glucose, and were managed using basal insulin. Women with pre-existing diabetes or who were later diagnosed with alternative forms of diabetes were excluded. The analysis data collection only focused on singleton pregnancies. Patients who did not adhere to the insulin titration protocol or who did not manage to record three consecutive fasting blood sugar demonstrating they have achieved target as defined above, we also excluded patients who had significant gaps in their insulin dosage records and could not reliably determine basal insulin levels at the time of target being achieved this via self-reported dose or healthcare professional notes on current dose. However, patients with partial missing data were retained where sufficient information was available to determine key outcomes, including time to achieve target fasting glucose and insulin dose requirements. This approach was taken to maximize use of clinically meaningful data while maintaining the validity and interpretability of outcome measures.

Clinical variables collected comprised demographic characteristics (maternal age at conception, ethnicity, parity, and gravidity), clinical measurements (height, pre-pregnancy weight, and pre-pregnancy BMI), lifestyle factors (smoking and e-cigarette use), OGTT values (fasting and 120-minute glucose values), capillary glucose monitoring data (captured electronically from the patient’s glucometer), glucose treatment (use of metformin and type and dose of basal insulin), pregnancy outcomes (mode of delivery including vaginal delivery, instrument delivery, elective or emergency cesarean section), and neonatal outcomes (birth weight, admissions to neonatal intensive care unit (ITU) or special care baby unit (SCBU), live births, Apgar score at 5 and 10 min). Pre-pregnancy weight and BMI were chosen as patients’ predictors for the models as they were routinely collected at booking appointment before insulin titration, providing a standardize baselines for all participants. All women were informed of the symptoms of hypoglycemia, advised to check their glucose during symptoms, and provided with treatment advice. We report the proportion of recorded glucose measurements during insulin treatment <3.5 mmol/L.

### Definitions of study outcomes

We assessed two key outcome measures of insulin requirement: the initial insulin requirement and the peak insulin requirement. Initial insulin requirement was defined as the basal insulin dose (units/day) required at the time the patient first achieved sustained (three consecutive days) fasting glucose below target (<5.3 mmol/L). Peak insulin requirement was defined as the highest basal insulin dose (units/day) used at any point during pregnancy.

We defined the time to target as the number of days between initiation of basal insulin and the first date on which fasting glucose values were consistently (three consecutive days) within target range (<5.3 mmol/L).

### Statistical analysis

Continuous variables were summarized using means and SD. Categorical variables were summarized using counts and percentages. Multivariable linear regression was used to assess associations between baseline characteristics (fasting OGTT result, 120-minute OGTT result, maternal age, and pre-pregnancy weight or pre-pregnancy BMI), and insulin requirements. Separate models were constructed for initial insulin requirement and peak insulin requirement as defined above. For each outcome, separate models were built with either pre-pregnancy weight or BMI given the collinearity between the two measures. Regression coefficients (β), 95% CIs, and p values were reported. Model assumptions were assessed visually using residual plots. R-squared was used to evaluate the proportion of variation in the outcome variable that the models explain, thereby assessing the model’s explanatory power. To compare relative variable importance, models were re-run with standardization of all continuous variables.

As a sensitivity analysis, to explore the potential modifying effect of concurrent metformin use, we re-ran the main analyses with the addition of a binary covariate for concurrent metformin use.

## Results

The study cohort consisted of 58 women with GDM who met our eligibility criteria (online supplemental figure S1). Their baseline characteristics are summarized in table 1. Pregnancy outcomes are presented in table 2, including mean time to target and proportion of women who required over 40 units of insulin to reach and maintain target fasting glucose. Across all participants, there were 6864 recorded capillary blood glucose measurements, of which 65 (0.90%) were below 3.5 mmol/L.

**Table 1:** Baseline characteristics of the women with gestational diabetes managed with patient-led basal insulin titration (n=58)

Variable	Mean±SD/n (%)
Demographics	
Maternal age at conception (years)	32.4±5.40
Ethnicity*	58 (100%)
Gravidity	2.5±1.6
Parity	0.9±1.1
Height (cm)	159.6±16.5
Pre-pregnancy weight (kg)	104.2±25.4
Pre-pregnancy BMI (kg/m²)	37.6±6.8
Smoking status	
Current smoker	6 (10.3%)
Current e-cigarette user	2 (3.4%)
Non-smoker†	50 (86.2%)
OGTT parameters	
Fasting OGTT (mmol/L)‡	5.6±0.9
2-hour OGTT (mmol/L)‡	8.2±2.2
Additional medication	
Metformin	34 (58.6%)
Mealtime insulin	12 (20.7%)
Basal insulin type	
Intermediate acting NPH	55 (94.8%)
Long-acting analog	3 (5.2%)

* White-British ethnicity.

† The non-smoker category includes ex-smokers.

‡ Diagnostic thresholds for gestational diabetes were fasting glucose ≥5.6 mmol/L and 2-hour glucose ≥7.8 mmol/L.

BMI, body mass index; NPH, Neutral Protamine Hagedorn; OGTT, oral glucose tolerance test.

**Table 2:** Pregnancy outcomes in women with gestational diabetes managed with patient-led basal insulin titration (n=58)

Outcomes	Mean±SD/n (%)
Glycemic outcomes	
Time from algorithm initiation to fasting blood glucose target (days)	10.5±11.8
Basal insulin units required to reach target	31.7±24.3
Required 40 units of basal insulin to achieve initial fasting glucose control	13 (22.4%)
Required ≥40 units basal insulin to maintain fasting blood sugar	32 (55.2%)
Highest dose of insulin units titrated up to maintain fasting blood sugars	50.8±33.2
Delivery outcomes	
Mode of delivery	
Vaginal delivery	24 (41.4%)
Instrumental vaginal delivery	5 (8.6%)
Emergency cesarean section	12 (20.7%)
Elective cesarean section	17 (29.3%)
Neonatal outcomes	
Birth weight (g)	3408±525
Neonatal ICU	1 (1.72%)
Neonatal SCBU	3 (5.17%)
Live births*	57 (98.3%)
Apgar 5 min	9.3±0.8
Apgar 10 min	9.6±0.5

* One fetal demise occurred; the stillbirth was classified as unexplained and not attributed to the GDM diagnosis.

GDM, gestational diabetes mellitus; ICU, intensive care unit; SCBU, special care baby unit.

Insulin requirements varied considerably within the cohort (figure 1). While some patients achieve control with the initial 14 units of basal insulin, others require substantial dose escalation, reflecting the variable degree of insulin resistance in patients with GDM.

**Figure 1:**
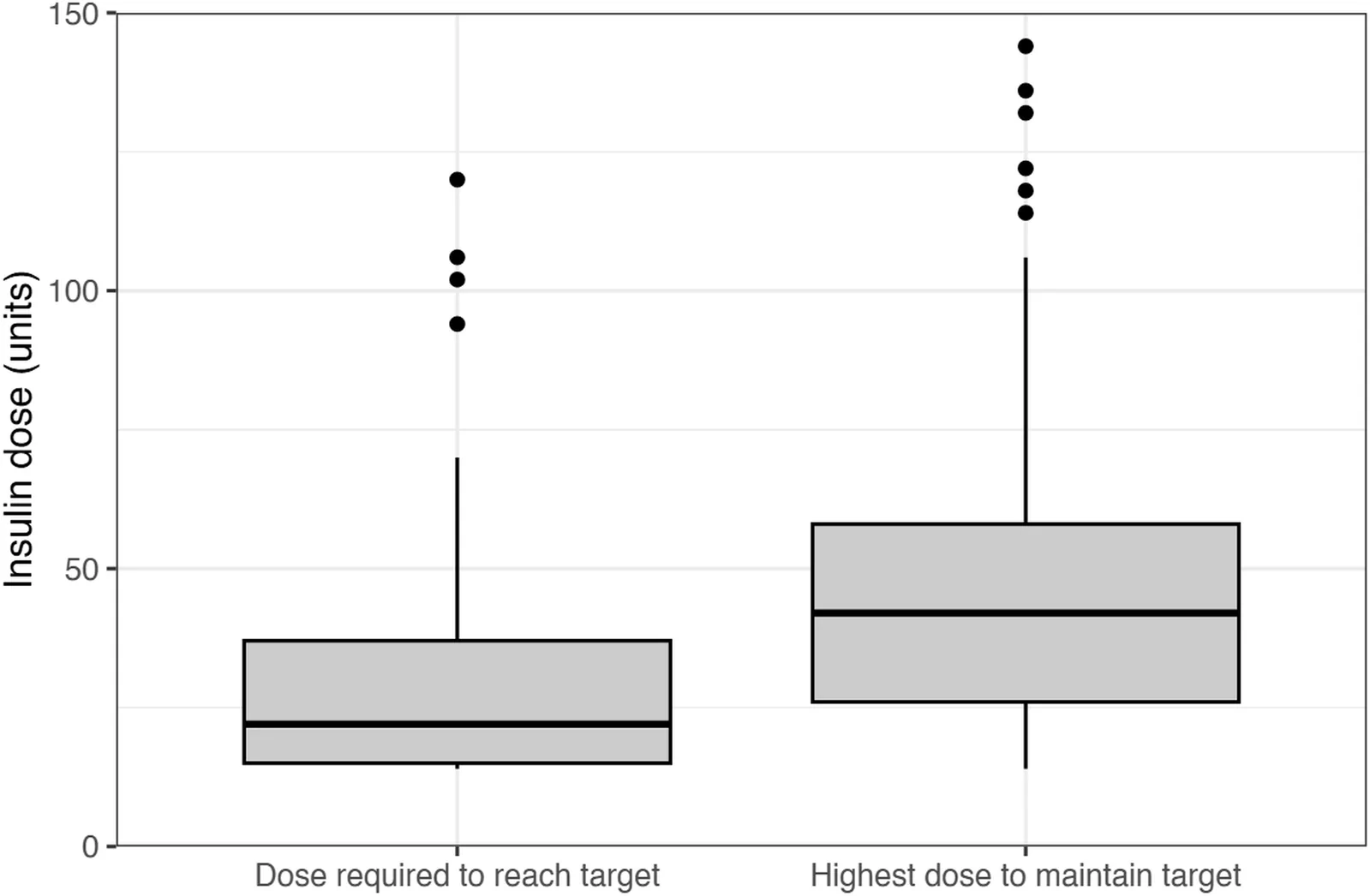
Boxplots of initial and peak basal insulin requirements. The horizontal line indicates the median, the box the IQR (25th–75th percentiles), whiskers extend to 1.5×IQR, and dots represent outlying observations.

Linear regression models demonstrated significant associations between OGTT fasting glucose, pregnancy weight (model 1) or BMI (model 2), and insulin dose required initially to achieve target, although performance of the model was limited (table 3). Similar associations were seen in models predicting maximum insulin (models 3 and 4; table 3); however, model performance was even lower than in those predicting initial insulin dose required to reach the target. The correlation between fasting and 120-minute OGTT glucose results and initial and peak insulin requirements are shown in online supplemental figures S2–S5.

**Table 3:** Linear regression models for initial and peak insulin requirements

	Initial insulin requirement (insulin requirement to get to treatment target)		Peak insulin requirement	
	Model 1: weight model β (95% CI; p value)	Model 2: BMI model β (95% CI; p value)	Model 3: weight model β (95% CI; p value)	Model 4: BMI model β (95% CI; p value)
Variables				
Maternal age (years)	0.7 (–0.1 to 1.5; 0.100)	0.6 (–0.3 to 1.4; 0.196)	0.3 (–1.2 to 1.8; 0.674)	0.2 (–1.2 to 1.7; 0.748)
OGTT fasting glucose (mmol/L)	17.4 (12.2 to 22.6; <0.001)	17.8 (12.1 to 23.4; <0.001)	15.5 (6.0 to 24.9; 0.002)	12.6 (3.1 to 22.0; 0.011)
OGTT 120-minute glucose (mmol/L)	1.2 (–0.9 to 3.4; 0.257)	0.9 (–1.3 to 3.1; 0.416)	−2.1 (–6.0 to 1.8; 0.289)	−2.1 (–5.8 to 1.6; 0.267)
Pre-pregnancy weight (kg)	0.2 (0.0 to 0.4; 0.022)	N/A	0.4 (0.1 to 0.7; 0.026)	N/A
Pre-pregnancy BMI (kg/m^2^)	N/A	0.4 (–0.3 to 1.2; 0.223)	N/A	1.9 (0.7 to 3.1; 0.003)
Model performance				
Model R-squared	0.61	0.58	0.30	0.36

BMI, body mass index; N/A, not applicable; OGTT, oral glucose tolerance test.

In models which compared the strength of variable association with insulin dose requirement via variable standardization (models 5 to 8; table 4), OGTT fasting glucose is the strongest predictor of initial insulin dose requirement, and OGTT fasting glucose, BMI, and pre-pregnancy weight had a similar strength of association with maximum insulin dose requirement. In sensitivity analyses, adjustment for metformin use did not substantially alter the relationships between OGTT glucose values, BMI, and insulin requirements (online supplemental table S1).

**Table 4:** Linear regression models for initial and peak insulin requirements with β coefficients standardized (z-scores); estimates represent the change in outcome (SD) per 1 SD increase in predictor

	Initial insulin requirement (insulin requirement to get to treatment target)		Peak insulin requirement	
	Model 5: weight model Standardized β (95% CI; p value)	Model 6: BMI model Standardized β (95% CI; p value)	Model 7: weight model Standardized β (95% CI; p value)	Model 7: BMI model Standardized β (95% CI; p value)
Variables				
Maternal age	3.8 (–0.7 to 8.3; 0.100)	3.0 (–1.6 to 7.6; 0.196)	1.7 (–6.5 to 9.9; 0.674)	1.25 (–6.5 to 9.0; 0.748)
OGTT fasting glucose	16.4 (11.5 to 21.3; <0.001)	16.8 (11.4 to 22.2; <0.001)	14.6 (5.7 to 23.5; 0.002)	11.8 (2.9 to 20.8; 0.011)
OGTT 120-minute glucose	2.7 (–2.0 to 7.4; 0.257)	2.0 (–2.9 to 6.8; 0.416)	−4.6 (–13.2 to 4.0; 0.289)	−4.6 (–12.7 to 3.6; 0.267)
Pre-pregnancy weight	5.4 (0.8 to 10.0; 0.022)	N/A	9.6 (1.2 to 18.0; 0.026)	N/A
Pre-pregnancy BMI	N/A	3.0 (–1.9 to 7.9; 0.223)	N/A	13.0 (4.8 to 21.2; 0.003)
Model performance				
Model R-squared	0.61	0.58	0.30	0.36

BMI, body mass index; N/A, not applicable; OGTT, oral glucose tolerance test.

## Discussion

Weight and BMI are poor predictors of the basal insulin dose required to achieve and maintain fasting blood glucose targets in patients with GDM. When adjusted for OGTT glucose results and maternal age, weight was only weakly correlated with initial insulin dose required (β=+0.2 units of insulin per kg; 95% CI 0.03 to 0.4; p=0.022) and with peak insulin dose required (β=+0.4 units of insulin per kg; 95% CI 0.05 to 0.7; p=0.026). This equates to an average additional initial basal insulin requirement of +2 units for every 10 kg increase in pre-pregnancy weight and an average additional peak insulin requirement of +4 units for every 10 kg increase in pre-pregnancy weight. When adjusted for OGTT glucose results and maternal age, BMI demonstrated no significant association with initial insulin requirement (β=+0.4 units of insulin per kg/m²; 95% CI −0.3 to 1.2; p=0.223) and a small association with peak insulin dose required (β=+1.9 units of insulin per kg/m²; 95% CI 0.7 to 3.1; p<0.01). Fasting glucose had the strongest association with initial insulin dose requirement and was similar to BMI for peak insulin dose requirement. Overall, model performance was very limited for insulin dose prediction.

These data demonstrate that current weight-based approaches to calculating initial basal insulin doses are likely to have limited clinical value and potentially serve only to overcomplicate insulin dose management. The large unexplained variability in insulin requirements observed within our cohort suggests that there are many uncaptured factors that determine insulin resistance in patients with GDM. We initially sought to develop a model capable of predicting an appropriate starting insulin dose based on patients characteristics, including pre-pregnancy weight or BMI, OGTT results, and maternal age. The substantial unexplained individual variation in insulin requirement among women with GDM suggests that calculating a precise starting insulin dose from these clinical features may not be an effective clinical strategy. Instead, our results support the use of a simple fixed starting insulin dose and the use of a rapid titration algorithm to get to target fasting capillary glucose. This approach allows insulin therapy to be individualized to the needs of the patients and respond dynamically to increased insulin resistance as the pregnancy progresses.

### Comparison with previous literature

Previous research shows that higher pre-pregnancy BMI, fasting glucose, and OGTT readings increase the likelihood of requiring insulin therapy^7–9^; however, these studies assessed only insulin initiation rather than dose requirements once initiated. Although our results also highlight an association between these factors and insulin dose requirement, they do not support the use of these variables as strong determinants of insulin dose. To the best of our knowledge, no previous study has attempted to predict insulin dose requirement in GDM.

### Strengths and limitations

A key strength of this study is the use of real-world clinical data from women with GDM who have used a rapid insulin titration protocol, which acts like a homeostatic mechanism to adjust basal insulin dose to meet their requirements. This has provided a unique dataset for the analysis of insulin requirement throughout pregnancy. Data collection on a small scale permitted high-fidelity capture of serial fasting glucose measurements and insulin dose adjustments throughout pregnancy, allowing for the evaluation of gestational insulin requirements over time, something not currently possible in large routinely collected datasets in the UK.

Several limitations should be considered. Our participants were from a single center and of white ethnicity only, which may limit how applicable our findings are to other healthcare settings or populations. The small sample size of the study may limit the power to detect weaker associations with insulin requirements. We did not have weight measurements available at the time of insulin start. Further work could explore whether this weight may be a better predictor of insulin dose requirement; however, given the very limited correlations we have seen, we believe this is unlikely to provide a substantial improvement over pre-pregnancy weight.

### Implications for clinical practice and future research

Our data suggest that weight-based or BMI-based selection of basal insulin doses in GDM is not an effective strategy and may overcomplicate management. Future research should aim to evaluate insulin dosing requirements in larger and more diverse cohorts.

## Conclusion

Weight and BMI are only weakly correlated with insulin requirement in GDM. Using a weight-based or BMI-based starting insulin dose cannot be recommended based on these data.

## Supplementary material

10.1136/bmjdrc-2026-006285 online supplemental file 1

## Data Availability

No data are available.
