# Supplementary material for: Association between weight/body mass index and insulin dose requirements in gestational diabetes: a retrospective cohort study
Source: BMJ Open Diabetes Res Care. 2026 Oct 13;14(5):e006285. doi: 10.1136/bmjdrc-2026-006285 (PMC13663699; doi:10.1136/bmjdrc-2026-006285)
Supplement: Supplementary file 1 [file bmjdrc-14-5-s001.pdf]

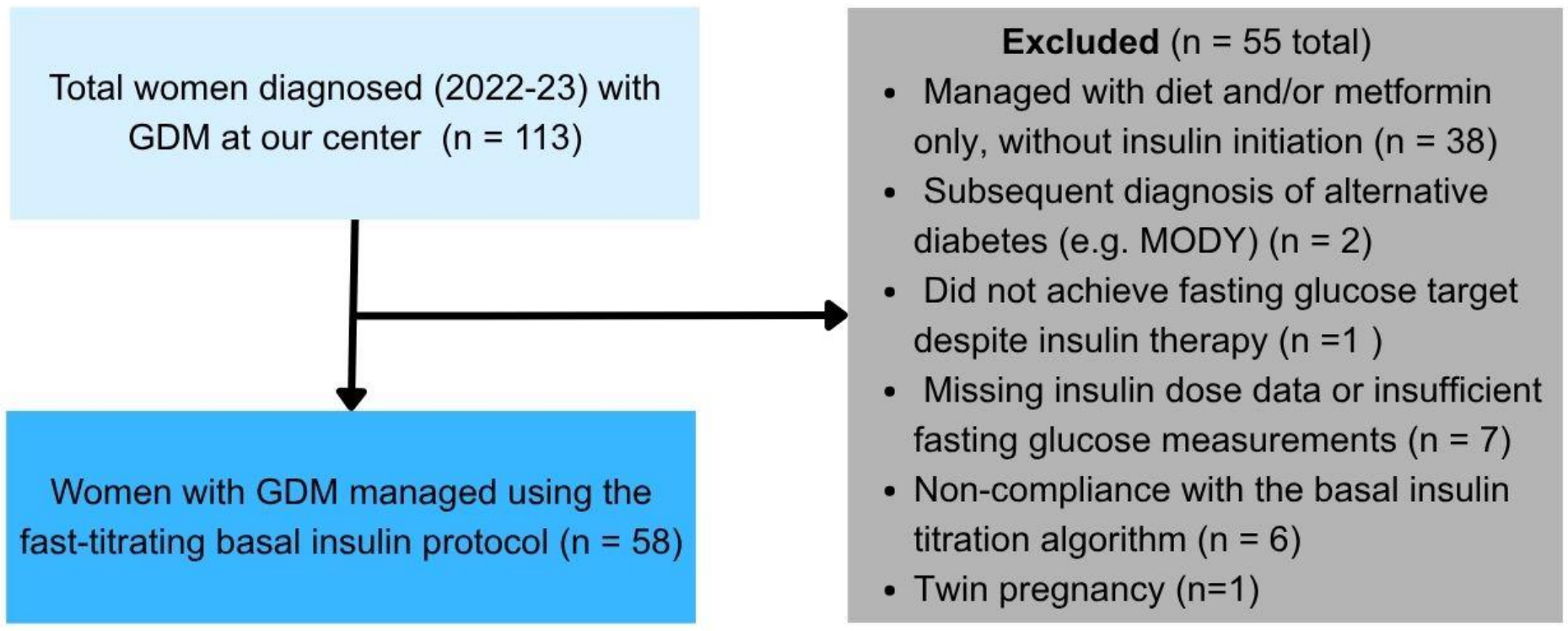

Figure S1. Study flowchart showing exclusions and derivation of the final cohort.

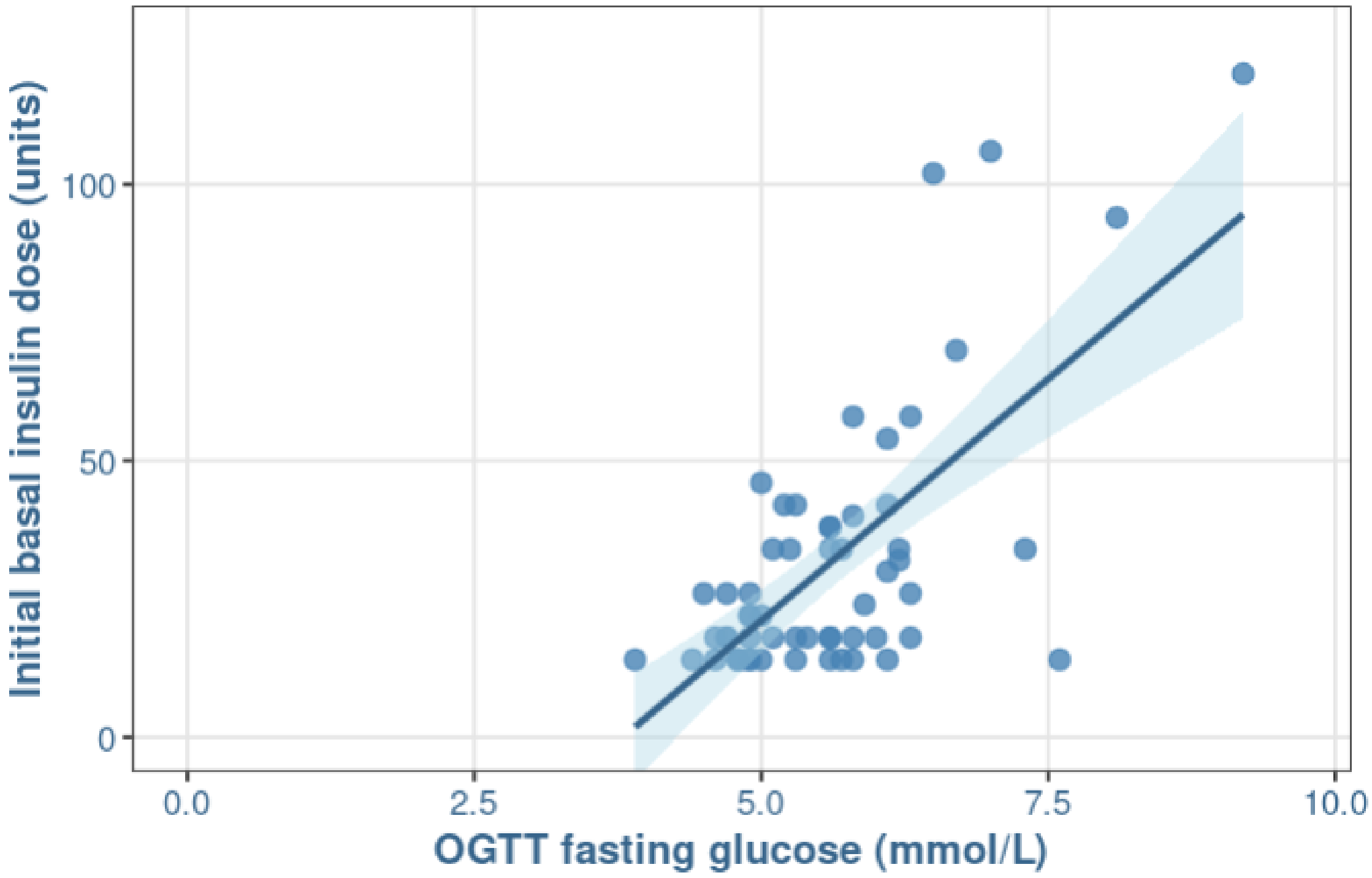

Figure S2. Scatter plot showing the relationship between fasting OGTT (mmol/L) and initial basal insulin dose (units) to reach target glycaemic control. Each point represents an individual participant. The solid line represents the fitted unadjusted linear regression line, and the shaded area represents the 95% confidence interval.

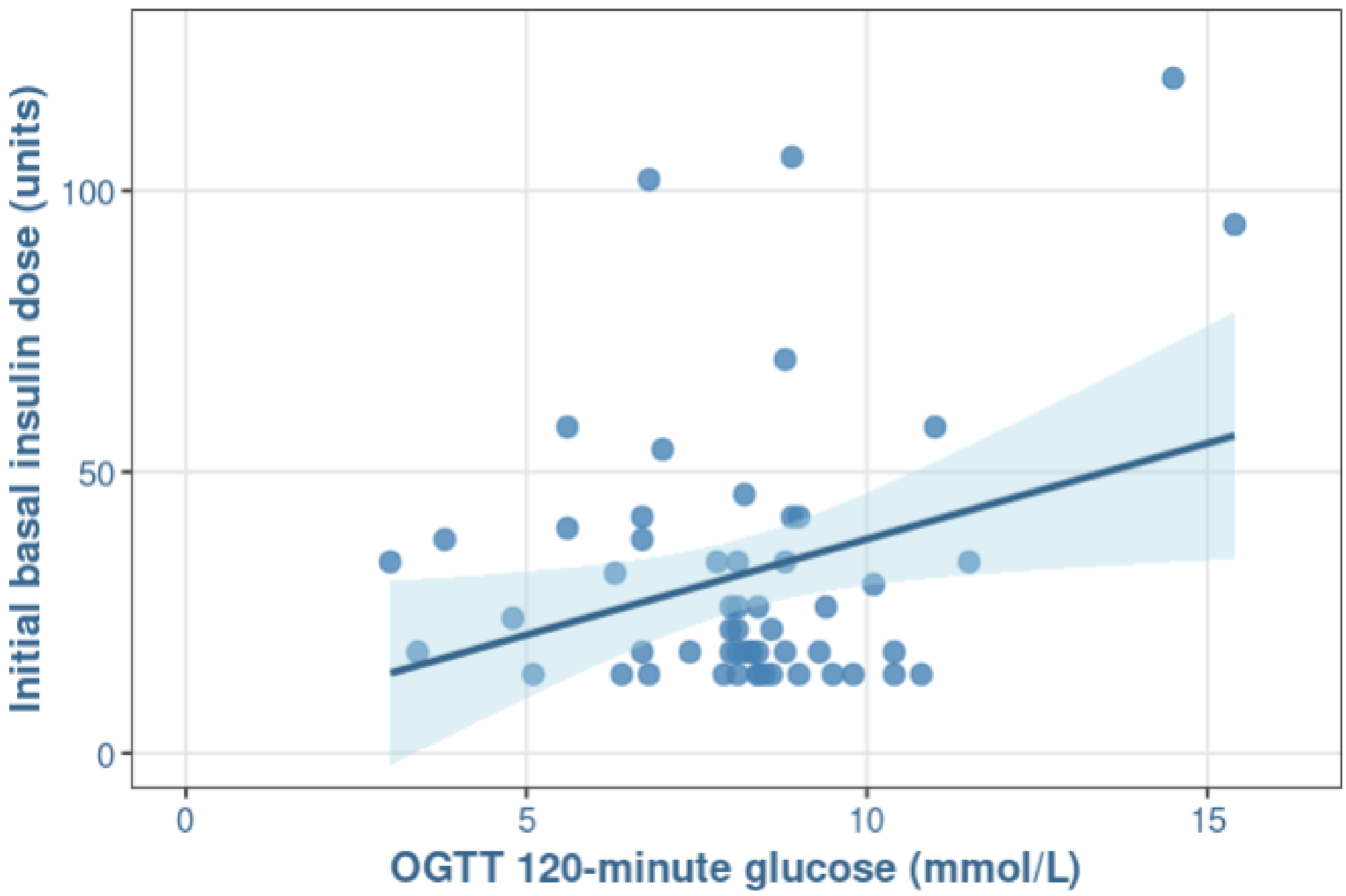

Figure S3. Scatter plot showing the relationship between 120 minutes OGTT (mmol/L) and initial basal insulin dose (units) to reach target glycaemic control. Each point represents an individual participant. The solid line represents the fitted unadjusted linear regression line, and the shaded area represents the 95% confidence interval.

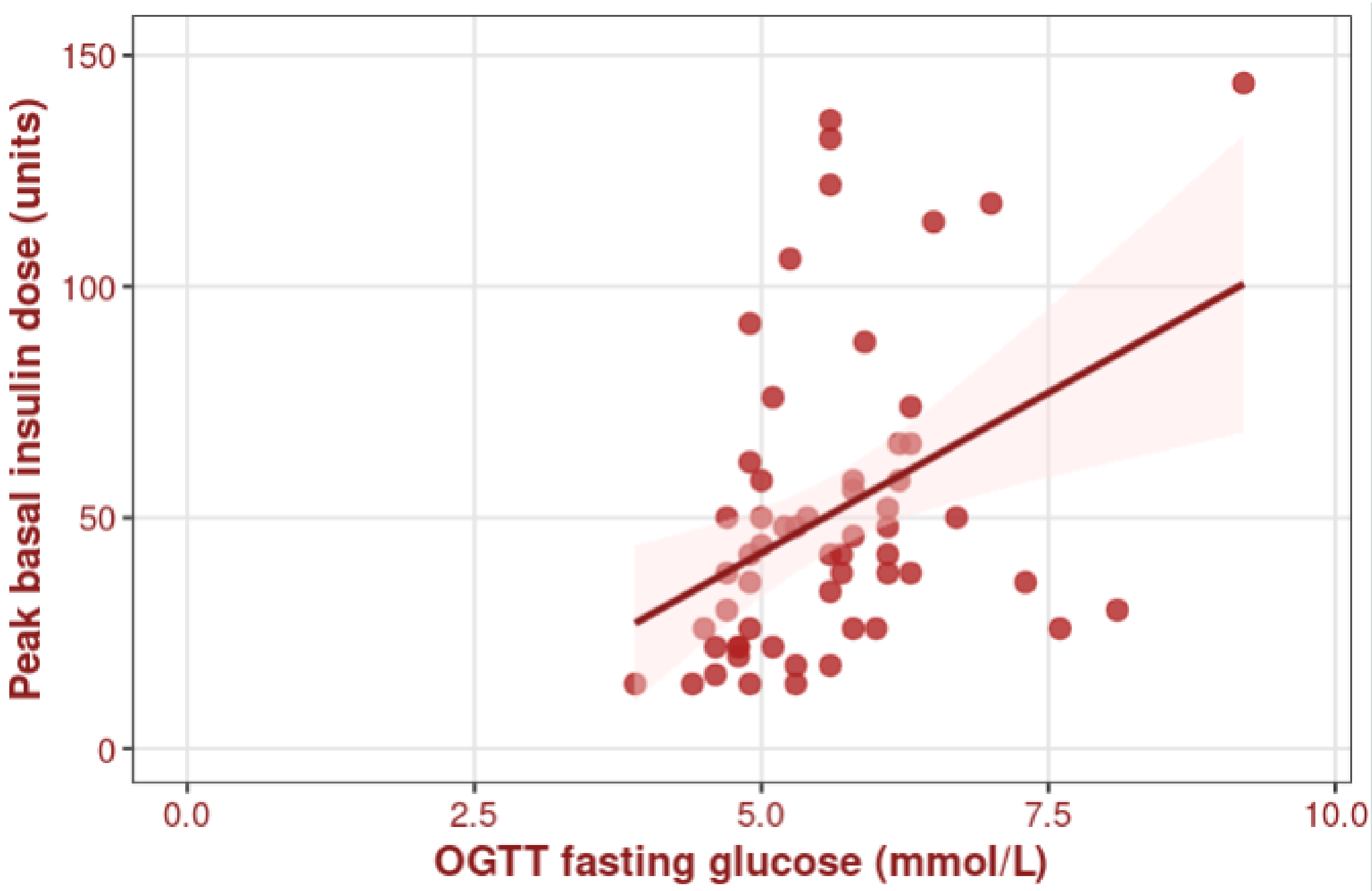

Figure S4. Scatter plot showing the relationship between fasting OGTT and peak insulin dosage (units) to maintain target glycaemic control. Each point represents an individual participant. The solid line represents the fitted unadjusted linear regression line, and the shaded area represents the 95% confidence interval.

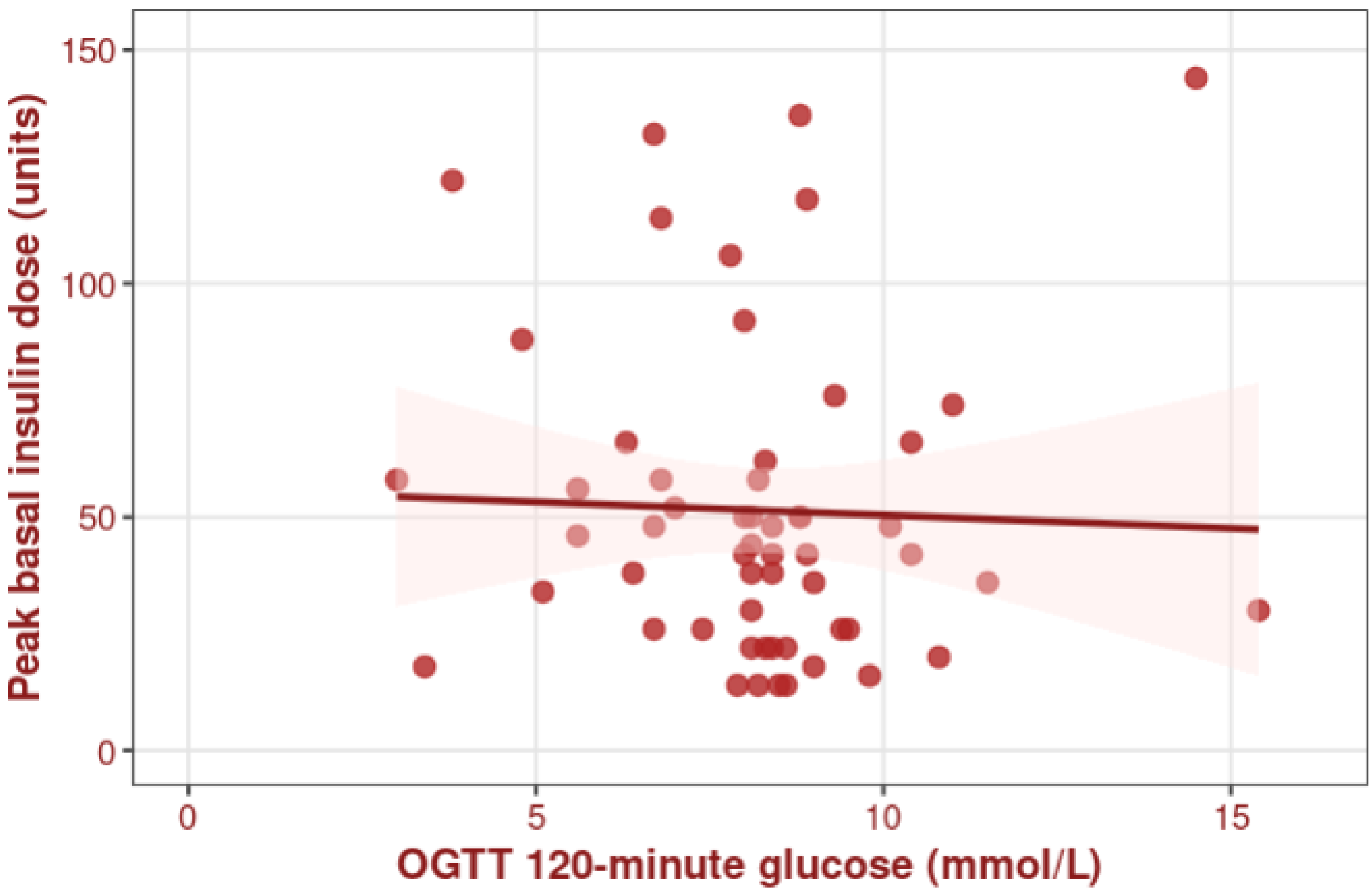

Figure S5. Scatter plot showing the relationship between 120 minutes OGTT and peak insulin dosage (units) to maintain target glycaemic control. Each point represents an individual participant. The solid line represents the fitted unadjusted linear regression line, and the shaded area represents the 95% confidence interval.

	Initial insulin requirement (insulin requirement to get to treatment target)		Peak insulin requirement	
	**Model 8: weight model** β (95% CI; p-value)	**Model 9: BMI model** β (95% CI; p-value)	**Model 10: weight model** β (95% CI; p-value)	**Model 11: BMI model** β (95% CI; p-value)
**Variables**				
Maternal age (years)	0.6 (-0.2, 1.5; 0.119)	0.5 (-0.3, 1.3; 0.239)	0.3 (-1.3 to 1.8; 0.731)	0.2 (-1.3, 1.6; 0.796)
OGTT fasting glucose (mmol/L)	18.7 (13.5, 23.9; <0.001)	19.4 (13.7, 25.1.; <0.001)	16.8 (7.1, 26.5; 0.001)	13.7 (3.8, 23.6; 0.008)
OGTT 120 minute glucose (mmol/L)	0.8 (-1.4, 2.9; 0.465)	0.4 (-1.8, 2.6; 0.699)	-2.5 (-6.5, 1.5; 0.207)	-2.4 (-6.2, 1.4; 0.211)
Pre-pregnancy weight (kg)	0.2 (0.02, 0.38; 0.027)	N/A	0.4 (0.03, 0.7; 0; 0.0317)	N/A
Pre-pregnancy BMI (kg/m2)	N/A	0.3 (-0.4, 1.0; 0.373)	N/A	1.8 (0.6, 3.0; 0.005)
Metformin use	9.3 (0.5, 18.1 ;0.040)	9.3 (-0.01, 18.6; 0.050)	9.3 (-7.2, 25.7; 0.265)	6.5 (-9.7, 22.6; 0.426)
**Model performance**				
Model R-squared	0.64	0.61	0.32	0.37

Table S1. Linear regression models for initial and peak insulin requirements with additional adjustment for concurrent metformin use. OGTT, oral glucose tolerance test; BMI, body mass index and additional metformin use; CI, confidence intervals.
